# Hydrological droughts in the southern Andes (40–45°S) from an ensemble experiment using CMIP5 and CMIP6 models

**DOI:** 10.1038/s41598-021-84807-4

**Published:** 2021-03-09

**Authors:** Rodrigo Aguayo, Jorge León-Muñoz, René Garreaud, Aldo Montecinos

**Affiliations:** 1 Centro EULA, Facultad de Ciencias Ambientales, Universidad de Concepción, Concepción, Chile; 2 Departamento de Química Ambiental, Facultad de Ciencias, Universidad Católica de la Santísima Concepción, Concepción, Chile; 3 Centro Interdisciplinario para la Investigación Acuícola (INCAR), Concepción, Chile; 4 Departamento de Geofísica, Facultad de Ciencias Físicas y Matemáticas, Universidad de Chile, Santiago, Chile; 5 Centro de Ciencia del Clima y la Resiliencia (CR2), Santiago, Chile; 6 Departamento de Geofísica, Facultad de Ciencias Físicas y Matemáticas, Universidad de Concepción, Concepción, Chile; 7 Centro de Recursos Hídricos para la Agricultura y Minería (CRHIAM), Concepción, Chile

**Keywords:** Climate sciences, Hydrology, Natural hazards, Ocean sciences

## Abstract

The decrease in freshwater input to the coastal system of the Southern Andes (40–45°S) during the last decades has altered the physicochemical characteristics of the coastal water column, causing significant environmental, social and economic consequences. Considering these impacts, the objectives were to analyze historical severe droughts and their climate drivers, and to evaluate the hydrological impacts of climate change in the intermediate future (2040–2070). Hydrological modelling was performed in the Puelo River basin (41°S) using the Water Evaluation and Planning (WEAP) model. The hydrological response and its uncertainty were compared using different combinations of CMIP projects (n = 2), climate models (n = 5), scenarios (n = 3) and univariate statistical downscaling methods (n = 3). The 90 scenarios projected increases in the duration, hydrological deficit and frequency of severe droughts of varying duration (1 to 6 months). The three downscaling methodologies converged to similar results, with no significant differences between them. In contrast, the hydroclimatic projections obtained with the CMIP6 and CMIP5 models found significant climatic (greater trends in summer and autumn) and hydrological (longer droughts) differences. It is recommended that future climate impact assessments adapt the new simulations as more CMIP6 models become available.

## Introduction

Anthropogenic climate change has increased the probability of extreme events in the mid-latitudes of the Southern Hemisphere, mainly those linked to severe droughts^1^. Projections indicate that these drought events may increase in extent, frequency and magnitude as they superimpose on the gradual decrease in precipitation^2,3^ and a significant increase in heat waves^4^. These changes have begun to be evident on the western side of the central Andes (31–38°S). This area has experienced an uninterrupted drought since 2010^5^, which has altered the spread of forest fires^6^, snow storage^7^, vegetation^5^ and nutrient input to coastal areas^8^, among other impacts.

During the last decades the Southern Annular Mode (SAM) has exhibited a clear trend towards its positive phase, attributed to stratospheric ozone depletion and increased greenhouse gases concentration^9,10^. This trend has favored dry conditions, mainly during the summer and autumn seasons (December to April) in the southern Andes^3^ (40–45°S). Dendrochronological reconstructions have shown that the present trend of the SAM is unprecedented in the last six centuries^11^, which has been evident in the precipitation and temperature of the southern Andes^12,13^. The trend towards a drier and warmer climate has also increased the climatic synergy with El Niño-Southern Oscillation (ENSO), promoting conditions of severe and extensive droughts^14^.

**Figure 1:**
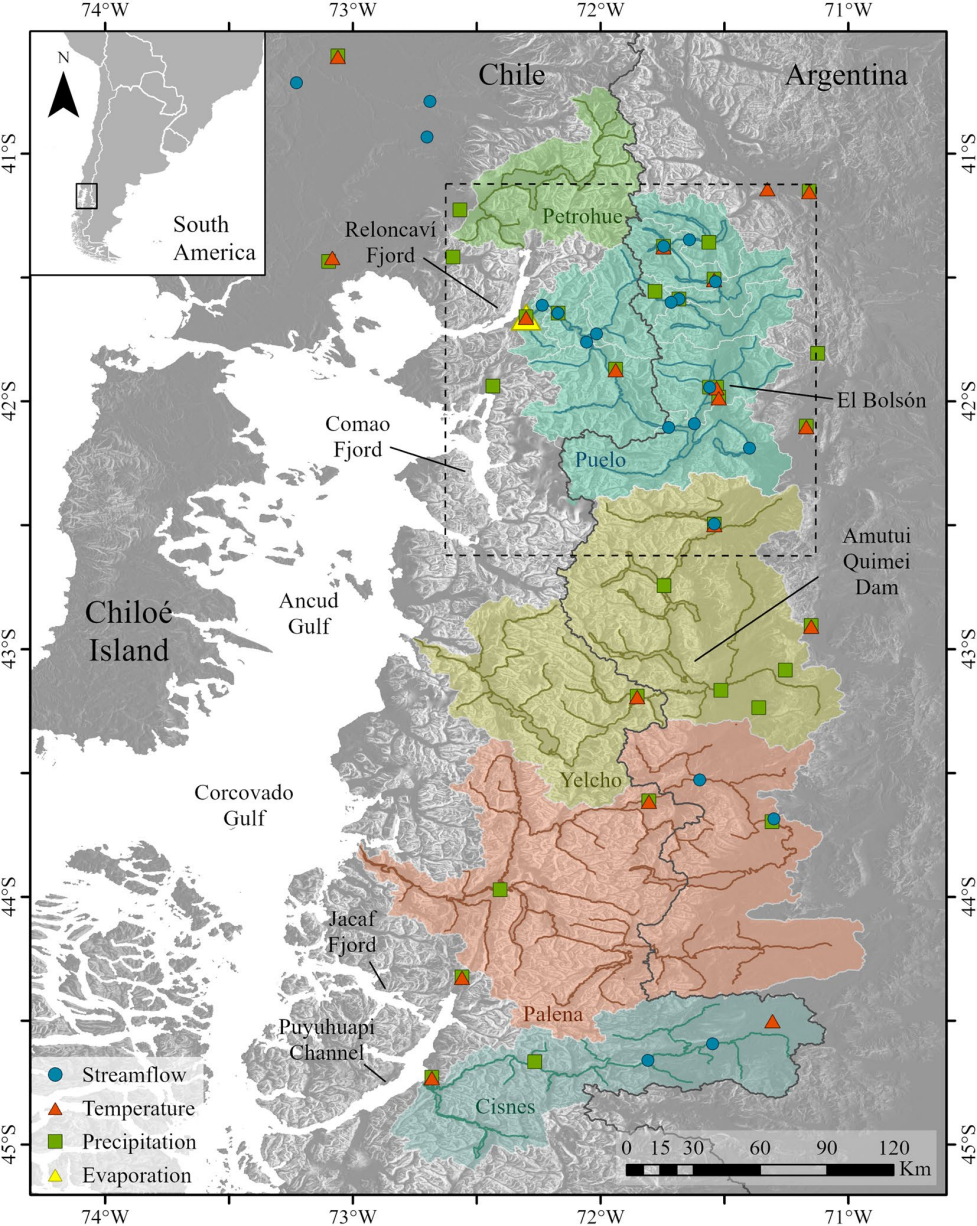
Study area. The colored areas indicate the selected basins and the symbols indicate the location of the meteorological and fluviometric stations. The subdivision in the Puelo River basin shows the sub-basins used in the hydrological modeling (“Hydrological model”). The dotted line indicates the area where the projections are downscaled (“Climate projections”). The figure was generated using ArcGIS Pro 2.7.0 (https://www.esri.com/en-us/arcgis/products/arcgis-pro/).

Although the western sector of the southern Andes still has large amounts of freshwater for human consumption, agriculture and hydroelectricity, variations in continental freshwater input can alter the land–ocean interface that sustains a complex estuarine system^15^. The freshwater enters the coastal area of the southern Andes through direct precipitation and surface runoff; the most important rivers are Petrohue, Puelo, Yelcho, Palena and Cisnes (Fig. 1). In these systems, freshwater generates a marked vertical stratification of two or three layers, products of strong density gradients largely dominated by variations in salinity^15^. The stratification of the water column is a key regulator of circulation patterns (e.g. water renewal rate) and primary production, limiting the depth of turbulent mixing^16^. The turbulent mixing determines the exchange of nutrients between the different layers of the water column, a process that can trigger pulses of primary productivity during spring and autumn and thus an increase of autotrophic biomass in the southern Andes inland seas^17–19^. Cuevas et al.^20^ determined that the structure of phytoplankton biomass varies directly with freshwater input and associated nutrient load, and to a lesser degree with surface solar radiation and photosynthesis.

The interaction among climate, hydrology and the physical–chemical characteristics of the coastal environment have been particularly evident in recent years along central-southern Chile. Masotti et al.^8^ estimated that the mega-drought in the central Andes reduced nutrient inputs to the ocean by 50%, with low levels of chlorophyll-a observed in the mouths of the main rivers in south-central Chile (33–38°S). The southern Andes has also witnessed droughts, although less persistent than farther north. During the year 2016, the coastal system received the lowest input of freshwater in several decades of instrument records. For example, the Puelo River, the main tributary of the Reloncaví Fjord (41°S), recorded the lowest streamflow in seven decades^21^ (Fig. 1). One of the consequences of this extreme condition was to have favored a record Harmful Algal Bloom (HAB), which produced losses of more than 800 million dollars in the aquaculture industry^21^, and a great social conflict due to the high employment that this industry provides^22^.

Climate projections in the Southern Andes suggest a prolongation of the dry conditions that have affected them over the past decades^12^. General Circulation Models (GCMs) from the Coupled Model Intercomparison Project (CMIP) phase 5 indicate a latitudinal pattern of decreasing precipitation that intensifies towards the central Andes, while temperature increases mostly on the eastern slopes of the Andes^12^. The new generation of CMIP6 models has improved in many aspects. For example, Bracegirdle et al.^23^ showed an overall reduction in the equatorward bias of the annual mean westerly jet from 1.9° in CMIP5 to 0.4° in CMIP6, which is clearer in austral spring and summer. This is accompanied by a halving of the bias of SAM decorrelation timescales compared to CMIP5. The results of CMIP6 models have shown greater climate sensitivity than previous simulations^24^, and therefore previous projections may be underestimated and need to be re-evaluated. As a result of these projections, in a region such as the southern Andes, where there is high seasonal snow storage, a more intense and extensive summer season is expected as a result of early snow melting^25^. Aguayo et al.^26^ estimated that the streamflow of the Puelo River (41°S, Fig. 1) could decrease by ~ 20% in summer and 15% in autumn by 2030–2060. The recurrence of extreme droughts (taking 2016 as a reference year) is also expected to double its probability, assuming that natural climate variability will be maintained in the future (delta change method).

The first objective of this study is to evaluate the effects of a future drier and warmer climate on the recurrence of severe droughts that could exceed critical thresholds of freshwater supply to the coastal-marine systems of the southern Andes (40–45°S) with detrimental impacts on land and marine ecosystems. A second objective is to analyze the uncertainty of the hydrological response to different combinations of CMIP projects (n = 2), climate models (n = 5), scenarios (n = 3) and univariate statistical downscaling methods (n = 3). The development of these objectives required the analysis of the main hydro-climatic trends from observational and satellite records, the evaluation of three univariate methods of statistical downscaling, and the projection of the hydrological impacts for the next five decades with the WEAP hydrological model in the Puelo River basin.

## Materials and methods

### Study area

The study area covers the southern Andes in northern Patagonia and the adjacent lowland and ocean sectors, from the Petrohue River Basin (~ 40°S) to the Cisnes River Basin (~ 45°S) (Fig. 1). The land geography includes numerous mountain basins dominated by native forest and a very irregular topography. The coastal area is composed of a system of fjords, bays and channels that is surrounded by mountains that reach 1000 m elevation just a few tens of kilometers from the coast (the main coastal systems are shown in Fig. 1). This narrow, intricate strip of land has a hyper-humid climate^14^ (> 3000 mm year^−1^) because weather systems embedded in the SH westerly wind belt arrive year-round and precipitation is further enhanced over the western slopes of the Andes^27^. At inter-annual timescales, the climate of southern Andes is disrupted by ENSO and SAM^28^. Under El Niño conditions there is a tendency for precipitation deficit^29^ in connection with a tendency for anticyclonic anomalies over the southern part of the continent that tend to block the incoming weather systems. Drier conditions prevail in southern Andes during the SAM positive phase due to the establishment of a circumpolar a ring of positive pressure anomalies over the SH midlatitudes that also hinders the arrival of weather systems^14,30^.

The hydrological modeling and downscaling process were performed in the Puelo River basin (41°S; Fig. 1), the main contributor of freshwater for salmon farming and mytilid cultures in the Reloncaví Fjord and Sound. The Puelo River Basin covers an area of ~ 9000 km^2^, of which 66% and 34% is in Argentina and Chile, respectively. The basin presents a high availability of hydro-climate information (Fig. 1), which has allowed good performance in previous hydrological modeling^26^. The Puelo River averages a streamflow (Q_m_) of 640 m^3^s^−1^ before its mouth in the Reloncaví Fjord, and like other rivers in the southern Andes, is characterized by its peaks in winter and spring^28,31^. Lara et al.^32^ showed that Puelo River streamflows are significantly correlated with other major rivers in the southern Andes (r > 0.4). The streamflow of the Puelo River has shown a decreasing trend during December-May during the last decades that is unprecedented in the last four centuries, according to dendrochronological studies^32^. Similarly, the Manso River (Q_m_ ~ 200 m^3^s^−1^), the main tributary of the Puelo River, has also shown a decreasing pattern over the last decades due, among other causes, to the progressive decrease of its headwater glaciers^33,34^. Associated with these trends, several studies developed in the Reloncaví Fjord have detected changes in the physicochemical properties of the water column (temperature, salinity and dissolved oxygen) and in the temporal patterns of primary productivity^19,31^.

### Data

The daily observed data of precipitation, temperature and streamflow for the period 1950–2019 were obtained from the General Directorate of Water (DGA) and the Meteorological Service (DMC) in Chile, and from the Undersecretary of Water Resources (SRHA) and the Meteorological Information Center (SMN) in Argentina (Fig. 1). Only stations with continuous records over the last years were considered. Following Wilby et al.^35^, a careful quality control of the daily data was performed. The data were then aggregated by month (only months with more than 20 records). In order to have spatially distributed data for the hydrological modelling process (“Hydrological model”), monthly data were complemented with estimates obtained from remote sensing data and atmospheric reanalysis models (Table 1).

**Table 1:** Summary of variables, sources and bias correction schemes for the hydrological modeling process (“Hydrological model”).

Variable	Source	Spatial resolution	Temporal resolution	Bias correction
Precipitation	CHIRPSv2	0.05°	Monthly	Scaling model between CHIRPSv2 and observations
Air temperature	MOD11C3	0.05°	Monthly	Statistical model considers the elevation and daytime/nighttime land temperature
Snow cover	MOD10A2 MYD10A2	500 m	8 days	Cloud filter^69^
Relative humidity	ERA5	0.25°	Monthly	Calculated from T and dew temperature from ERA5
Wind speed	ERA5	0.25°	Monthly	Logarithmic wind profile and transfer function according to observations

More information on bias correction can be found in Aguayo et al.^26^. All bias correction schemes were performed in the period 2000–2019.

The land cover was obtained from a Landsat satellite image (year 2001), previously corrected and classified following the methodology proposed by Fuentes et al.^36^. The precision of the classifications was evaluated using confusion matrices constructed using data extracted from the Cadaster of Native Forests of Chile and training points obtained from different field campaigns. Finally, the land cover was classified into forest, shrubland, grassland, bare soil, snow or ice and water bodies (Supplementary Fig. S1).

### Hydrological model

**Figure 2:**
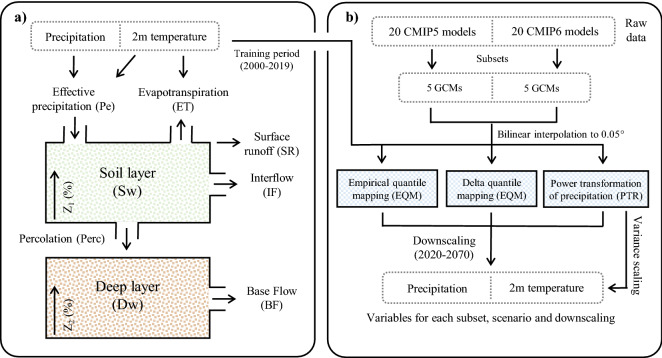
(**a**) Conceptual scheme of the hydrological WEAP model. (**b**) Downscaling methodology of climate projections CMIP5 and CMIP6.

The hydrological Water Evaluation and Planning model^37^ (WEAP) was selected to project the hydrological impacts over the next decades. The WEAP model has demonstrated good performance throughout Chile and has been the most used for climate change studies in the central Andes (30–38°S; Table 2). The WEAP model is a semi-distributed model that represents the relevant hydrological processes in a one-dimensional conceptual model of two storages (soil moisture method; Fig. 2a). Potential evapotranspiration is calculated with the Penman–Monteith equation, while snowmelt is modeled with the degree-day method. Other relevant processes are represented by empirical functions that describe surface runoff, interflow and percolation, as a function of soil layer and deep layer storage (Fig. 2a).

**Table 2:** Climate change impacts on the Andes hydrology of Chile.

Basin or area	Latitude	Hydrological model	Climate models	Downscaling	References
Vergara and Lonquimay	38°S	SWAT	1 RCM + 7 GCMs	Delta change	Stehr et al.^70^
South-central Chile	33–38°S	WEAP	1 GCM	Transfer function	McPhee et al.^71^
Limarí	31°S	WEAP	1 RCM	–	Vicuña et al.^59^
Limarí	31°S	WEAP	20 GCMs	Delta change	Vicuña et al.^72^
Mataquito	35°S	VIC	12 GCMs	EQM	Demaria et al.^58^
Maipo	33°S	WEAP	3 GCMs	PQM	Meza et al.^73^
Chile	19–56°S	VIC	5 GCMs	QDM	DGA^53^
Central Chile	34–37°S	VIC	19 GCMs	PQM	Bozkurt et al.^2^
Puelo	41°S	HBV	25 GCMs	Delta change	Aguayo et al.^26^
Traiguén	38°S	WEAP	1 RCM	Delta change	McNamara et al.^74^

*EQM* empirical quantile mapping, *PQM* parametric quantile mapping, *QDM* quantile delta mapping.

The WEAP input variables are precipitation, temperature and (optionally) relative humidity and wind speed. Given the low availability and spatial representation of available observation records for the southern Andes^26,38^, the corrected products of Table 1 were used. The modeling considered a monthly time step, a time window adequate for the characterization of hydrological droughts^39^. The modeling considered the subdivision of the Puelo River basin into nine sub-basins, each delimited according to fluviometric stations representative of the river network (Fig. 1 and Supplementary Fig. S1). In parallel, each sub-basin is divided into elevation bands with variable ranges (300–1200 m). The ranges were defined according to the basin hypsometry, in order to distribute the bands in similar areas. Finally, for each elevation band, the equations of the hydrological model were solved based on the land cover shown in Supplementary Fig. S1.

The model calibration used the Parameter Estimation and Uncertainty Analysis^40^ method for the period 2000–2010 (water year April-March), based on the parameters and methodologies detailed in Supplementary Table S1 and Supplementary Text S1, respectively. In addition, the simulated values of Snow Cover Extent (SCE) and Potential Evapotranspiration (PET) were compared using MODIS satellite (Table 1; 2002–2019) and evaporation data (Fig. 1; 2002–2012), respectively. Once the calibration stage was completed, the model was validated for the period 2011–2019 (water years). It should be noted that three sub-basins began operating in 2009 (ALP, FM and PF, Supplementary Fig. S1), so their calibration and validation was performed for the 2009–2014 and 2015–2019 periods, respectively. Considering that the simulation of a drying climate requires a more balanced consideration of the mid-low streamflows, the Modified Kling-Gupta Efficiency (KGE) and the Refined Index of Agreement (RIA) were used as performance indicators in both stages. The KGE index squares the error in each time step. In contrast, the objective of RIA is to minimize the sum of absolute errors^41^.

### Climate projections

Climate projections of monthly precipitation and temperature were obtained from 40 General Circulation Models (GCMs) from the CMIP5 and CMIP6 projects (20 GCMs per project; Supplementary Table S2). Each model considered only one output, preferably rlilp1 and r1i1p1f1 for CMIP5 and CMIP6, respectively. The models were classified according to the scenarios SSP126, SSP245, SSP585 (SSP; Shared Socioeconomic Pathway), RCP2.6, RCP4.5 and RCP8.5 (RCP; Representative Concentration Pathway). Most of the selected models prescribe the ozone values (NOCHEM); the remaining ones have interactive ozone using a coupled chemical climate model^10^ (CHEM; Supplementary Table S2).

Considering that several studies have identified climate models and their scenarios as the major source of uncertainty^42^, five GCMs were selected for each project (Fig. 2b; Supplementary Table S2). The selection of ten GCMs can ensure that the median of different combinations generates similar uncertainty components as the whole ensemble^43^. The models were selected according to the skill in reproducing past climate in the region. This evaluation was done by Bozkurt et al.^44^ and Rivera and Arnould^45^ for the CMIP5 and CMIP6 projects, respectively. In addition, it was verified that the 10 GCMs selected represented the full range of climate projections generated from the 40 GCMs, and different levels of climate sensitivity.

Three univariate statistical downscaling methodologies were evaluated for each subset to assess the differences between the methods and their subsequent impact on the hydrological projections (Fig. 2b). The downscaling reduces the resolution of the GCMs to a hydrologically adequate resolution (0.05°) that allows capturing the spatio-temporal variability of precipitation and temperature in Table 1. The selected methods were: Empirical Quantile Mapping (EQM), Quantile Delta Mapping (QDM) and Power Transformation of Precipitation (PTR) (Fig. 2b).

EQM has been widely used^46^, and consists of calibrating the simulated cumulative distribution function by adding to the observed quantiles both the mean delta change and the individual delta changes in the corresponding quantiles. In contrast, QDM multiplies observed values by the ratio of the modeled values in the same quantiles^47^. Finally, PTR adjusts the variance statistics of precipitation time series in an exponential form^48^. The power parameter is defined by matching the coefficient of variation (CV) of corrected monthly simulated precipitation with the CV of observed monthly precipitation. Note that PTR is only applicable to precipitation, so a variance and mean temperature scaling is generated (Fig. 2b). All the methods were done with the Climate4R library developed by Iturbide et al.^49^, and consider the periods 2000–2019 and 2020–2070 as baseline and projection periods, respectively. The baseline period was chosen to maintain temporal consistency with the hydrological modeling. The different combinations of CMIP projects (n = 2), climate models (n = 5), scenarios (n = 3) and univariate statistical downscaling methods (n = 3) generated 90 scenarios of precipitation and temperature conditions (relative humidity and wind speed remain constant) that were evaluated in the WEAP model (“Hydrological model”).

### Statistical analysis

#### Seasonal trends

The significance of the seasonal historical trends of precipitation, temperature, streamflow and snow cover was analyzed with Mann–Kendall tests. In order to increase spatial representativeness, this analysis considered all stations whose instrumental records were greater than 25 years in the period 1950–2019 (without gap filling). The period of snow cover evaluated was 2002–2019. In parallel, the Indicators of Hydrologic Alteration^50^ and the water year hydrograph centroid^51^ were estimated for the station located at the mouth of the Puelo River (Fig. 1). This station has the most extensive records in the basin, and possibly the longest in Western Patagonia (water years 1950–2019; < 1% of missing data). Other long-term fluviometric records were not analyzed due to their location downstream from dams (Amutui Quimei Reservoir in Fig. 1).

#### Hydrological impacts

The hydrological impacts produced by the 90 climate scenarios could be reflected in the Puelo River hydrology at different time scales. For this reason, different approaches to comparison were evaluated. To identify seasonal changes on the hydrological regime, the *first approach* compared average monthly streamflow in two time periods (2000–2019 *vs*. 2040–2070). The *second approach* analyzed changes in the frequency of minimum annual streamflows considering moving time windows of one, three and six months. This analysis used a Weibull distribution, due to its theoretical base, the fact that it has a lower bound and its popularity for low-flow studies^52^. The *third approach* evaluated the projections of hydrological droughts that could produce impacts in the coastal-marine system of the southern Andes (hereinafter severe droughts). This analysis considered the frequency, duration and hydrological deficit (volume) of streamflows lower than 250 m^3^s^−1^ (95% probability of non-occurrence). Below this threshold, anomalous oceanographic events have been reported in the Reloncaví Fjord, such as hypoxia events and HABs^19,21,31^. In the first and third approaches, the significance of the differences produced by different CMIP projects and methodologies were evaluated with the Mann–Whitney-Wilcoxon non-parametric test (MWW test).

## Results

### Observed spatio-temporal patterns

**Figure 3:**
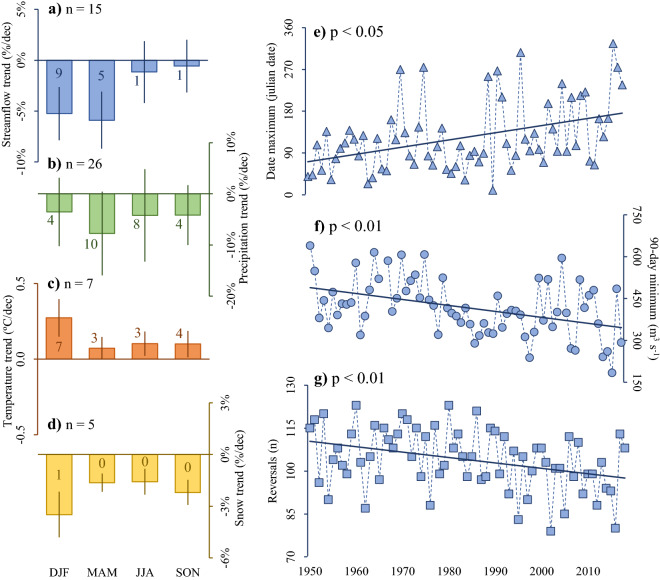
Hydroclimatic trends in the southern Andes. The panels on the left represent Sen's slope (> 25 years in the period 1950–2019) of streamflow (**a**), precipitation (**b**), temperature (**c)** and snow cover (**d**; 2002–2019). Each mean trend is accompanied by the number of stations or basins with significant trends (p < 0.05). The panels on the right correspond to Indicators of Hydrologic Alteration for the mouth of the Puelo river (**e**–**g**; Fig. 1). The error bands indicate the standard deviation according to the total number of stations or basins (n).

The historical trends in the period 1950–2019 (> 25 years) vary seasonally depending on the variable analyzed (Fig. 3). Consistent with previous studies^3^, precipitation over the study area has shown a decline year-round, greater in autumn (− 8 ± 8% per decade; Fig. 3b). Although autumn contributes 25% of annual precipitation (Supplementary Fig. S2), a significant number of stations had significant trends during winter (47% of annual precipitation). In contrast, air temperature showed a significant increase in all seasons, with the greatest warming in summer months (+ 0.3 ± 0.1 °C per decade; Fig. 3c). All the stations analyzed presented significant trends in this period. The trends show a heterogeneous pattern, with the eastern sector recording warming differences of 0.15 ± 0.04 °C per decade in relation to the western sector. For example, during the summer the western and eastern sectors showed values of 0.4 °C and 0.2 °C per decade, respectively.

**Figure 4:**
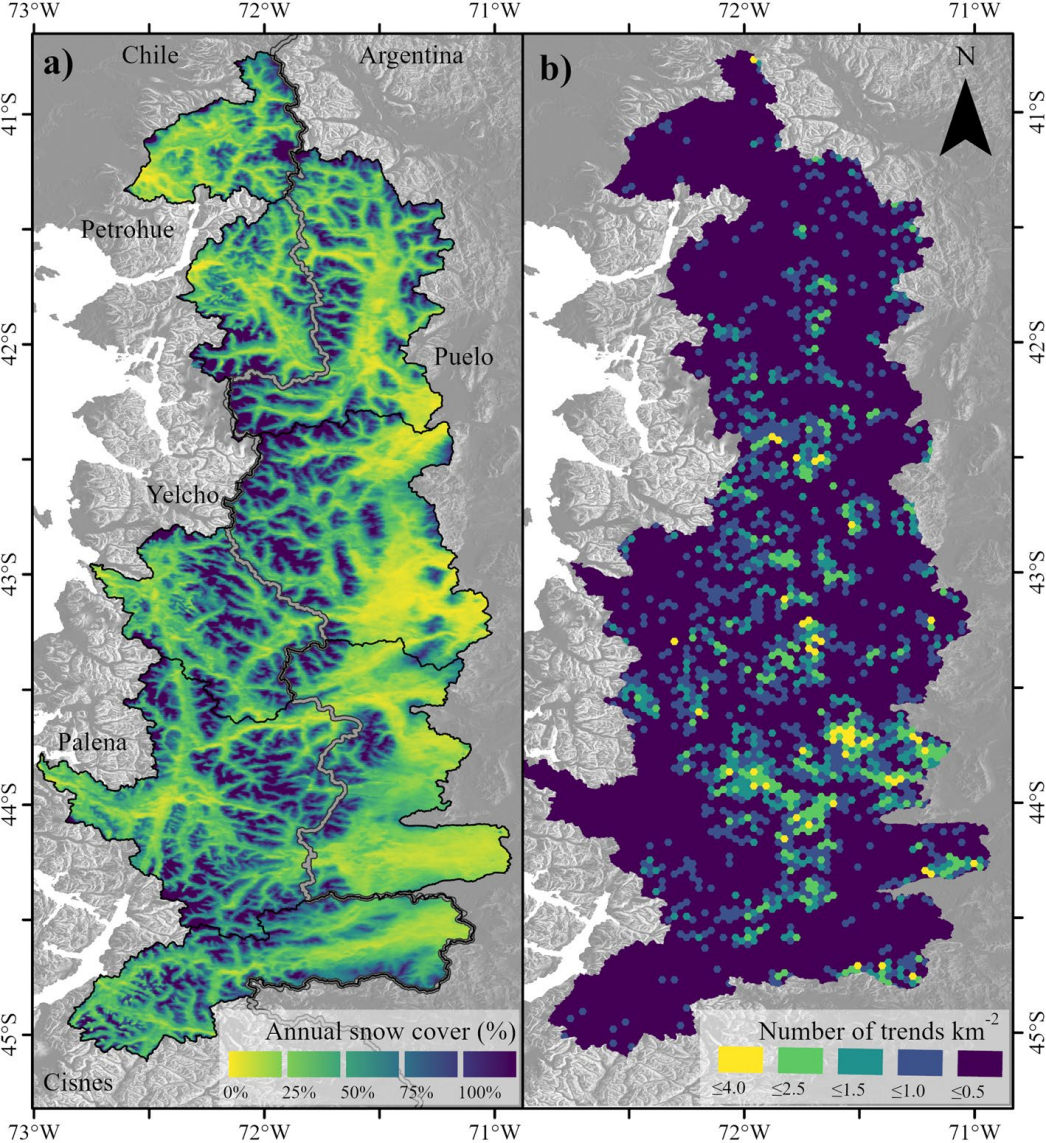
Snow cover in southern Andes. (**a**) Mean snow cover for the period 2002–2019. (**b**) Number of cells with significant decreasing trends per km^2^ in the same period (p < 0.05). The figure was generated using ArcGIS Pro 2.7.0 (https://www.esri.com/en-us/arcgis/products/arcgis-pro/).

The precipitation and air temperature trends were reflected in the snow cover (period 2002–2019), which had a decreasing trend during the entire year (Fig. 3d). Although the greatest trends were concentrated during the summer season (− 3 ± 1% per decade), only the Puelo River basin showed a statistically significant trend (p < 0.05; Fig. 3d). The spatial trends were generated mainly on the eastern slope of the Andes towards Argentina (Fig. 4), where consistently higher temperature increases and lower snow cover were detected. This pattern intensifies towards the south, with the Yelcho and Palena River basins being the most affected (Fig. 4b).

Changes in climate and snow cover ultimately result in surface hydrology trends. Puelo River streamflow trends were observed mainly in summer and autumn (Fig. 3a); the autumn period was the most affected with a trend of − 6 ± 3% per decade. However, the highest number of stations with significant trends (p < 0.05) was recorded in the summer (Fig. 3a). Similarly, the Indicators of Hydrologic Alteration recorded significant trends in several components of the natural flow regime, in timing (date of maximum streamflow; Fig. 3e), magnitude (minimum streamflow in 90 days; Fig. 3f) and rate of change (reversals; Fig. 3g). In contrast to the date of maximum streamflow, the water year hydrograph centroid did not show a statistically significant trend.

### Hydrological modelling

**Figure 5:**
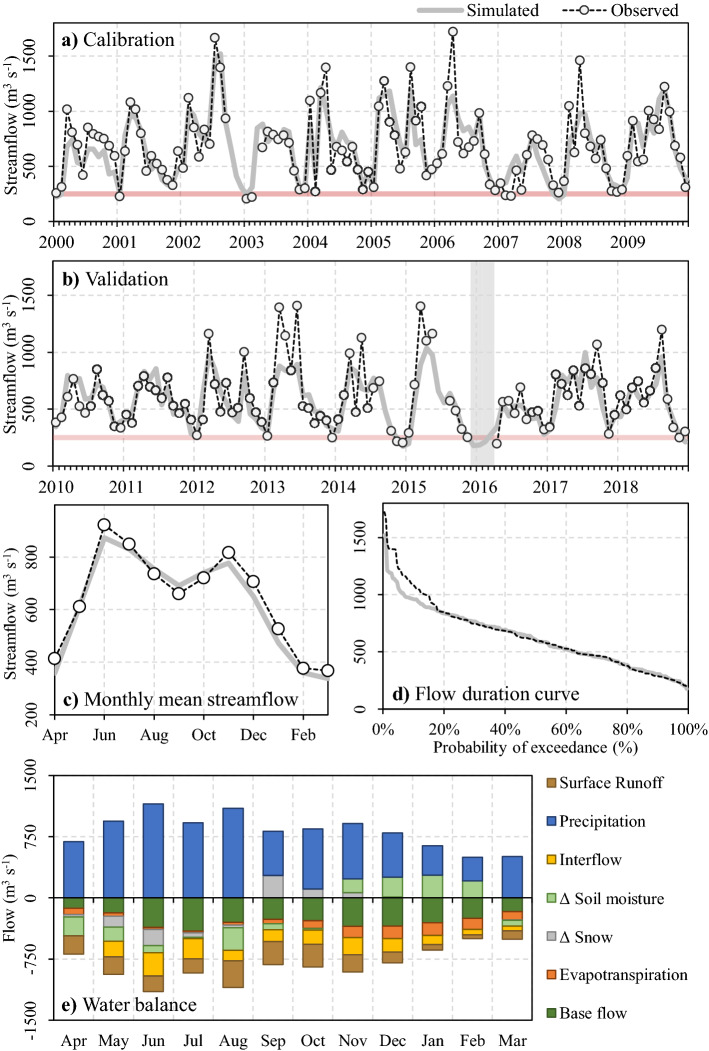
Performance of the hydrological WEAP model during the calibration (**a**) and validation (**b**) stages for the sub-basin located near the mouth of the Puelo River in the Reloncaví Fjord (PD in Supplementary Fig. S1). The horizontal line indicates the 250 m^3^s^−1^ threshold defined for severe droughts. (**c**) Comparison of monthly mean streamflow (sub-basin PD). (**d**) Flow duration curve (sub-basin PD). (**e**) Water balance for the Puelo River basin (period 2000–2019).

The WEAP hydrologic model obtained average performance values greater than 0.7, adequately capturing the streamflow regime of the Puelo River basin (Fig. 5). In the sub-basin located near the mouth of the river (PD in Supplementary Fig. S1), the model achieved a performance of 0.83 and 0.76 for the KGE and RIA indices, respectively (calibration period in Fig. 5a). The simulated average monthly streamflows in Fig. 5c had a dry bias associated mainly with high flow events, when the probability of exceedance was less than 20% (Fig. 5d). In contrast, low flows (Fig. 5d), minimum annual flows (R^2^ = 0.71), and attributes of severe drought events (frequency, duration and hydrological deficit) were adequately simulated. Despite the difference in the consideration of low and medium flows, the RIA and KGE indices converged to similar performance values in the calibration and validation stage (Supplementary Fig. S3).

When the results were disaggregated by sub-basin, the highest performances were achieved in the sub-basins located at low altitudes (MP, PM and PD in Supplementary Fig. S3), while the lowest performances were found in the sub-basins that presented lower temporal extension of flows (ALP, FM and PF in Supplementary Fig. S3). According to the KGE index, the lower performance obtained during the validation stage was the result of lower correlations (r = 0.78 ± 0.07), higher dry biases (β = 0.93 ± 0.11) and a lower capacity to capture streamflow variability (γ = 0.82 ± 0.08).

The baseline of the hydrological balance highlighted precipitation as the main driver of local hydrology (Fig. 5e). This influence was reduced during the summer months, when evapotranspiration can reach 40% of the total precipitation. Despite the limitations of relative humidity and wind, the simulated PET values were similar to those recorded by the evaporation pan (Root-Mean-Square Error (RMSE) = 14 mm, R^2^ = 0.87; Supplementary Fig. S3c). Snow accumulates during the months of May and August, and subsequently melts during September and November (reaching a peak in September; Fig. 5e). The results of the validation of SCE were reasonable (RMSE = 16%, R^2^ = 0.88). However, the simulated values tended to underestimate (overestimate) the SCE during the summer (winter) (Supplementary Fig. S3d). The streamflow composition (base flow, interflow and surface runoff) also showed important seasonal variations (Fig. 5e). For example, during the summer, baseflow contributed 61%, while surface runoff only 16%. During these months, soil moisture depletion represents an important input to the water balance (Fig. 5e).

### Raw climate projections

Regardless of the scenario chosen, climate projections for the period 2040–2070 obtained from 20 GCMs of the CMIP6 project suggest a prolongation of the drying and warming that have affected the southern Andes (“Observed spatio-temporal patterns”), using the period 2001–2018 as a reference (Supplementary Fig. S4). The decrease in precipitation follows a latitudinal pattern, with the greatest decrease in the northern part of the study area (Supplementary Fig. S4). In contrast, temperature increases follow a longitudinal pattern; the largest increases are concentrated east of the southern Andes (Supplementary Fig. S4).

**Figure 6:**
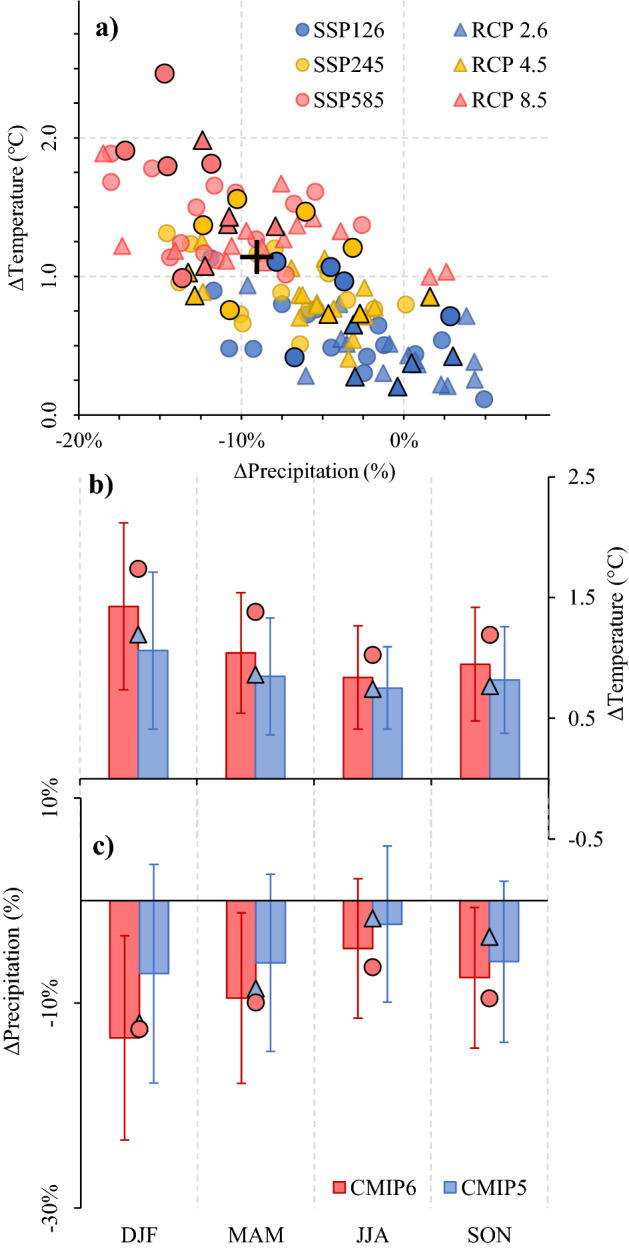
Raw climate projections of precipitation and temperature for the period 2040–2070 obtained from the CMIP5 and CMIP6 projects in the Puelo River basin (Fig. 1). Panel (**a**) indicates the mean annual rates of change. The black borders indicate the 5 GCMs selected for each CMIP project. The cross indicates the multi-model mean of the GCMs used in the National Water Balance for Chile^53^ (RCP 8.5; 1985–2015 *vs.* 2030–2060). Panels (**b**) and (**c**) show the seasonal change rates of precipitation and temperature, respectively, considering all scenarios (RCP 2.6, 4.5 and 8.5 for CMIP5 and SSP 126, 245 and 585 for CMIP6). The circles indicate the multi-model averages of the 5 GCMs. All projected changes consider the period 2001–2018 as a reference. The error bands indicate the standard deviation according to the total number of models.

The spatial and seasonal patterns projected by the CMIP6 models were similar to the CMIP5 counterparts (Fig. 6b,c). However, the trends projected towards the period 2040–2070 were significantly higher during summer and autumn, using a 95% confidence level (Fig. 6; Table 3). Considering two scenarios with similar radiative forcing, the SSP 585 multi-model mean projected temperature increases of 2.1 °C ± 0.5 °C in the Puelo River basin (DJF period), while the RCP 8.5 counterparts projected 1.7 °C ± 0.5 °C. The projected decreases for precipitation in the same season were − 20 ± 9% and − 11 ± 11% for the CMIP6 and CMIP5 projections, respectively. These values are slightly higher than those used by DGA^53^ (Fig. 6a). On the other hand, the projected changes in the frequency of climate anomalies did not follow the projected average trends. In this case, the variability, reflected in the standard deviation of the anomalies, remained constant (Supplementary Fig. S5).

**Table 3:** Results of the Mann–Whitney-Wilcoxon test between different CMIP projects (95% confidence level).

Comparison	CMIP5 *vs.* CMIP6		
Variable	Precipitation	Temperature	Streamflow
DJF	p < 0.001	p < 0.01	p < 0.001
MAM	p < 0.05	p < 0.05	p < 0.05
JJA	p < 0.1	NSC	p < 0.1
SON	NSC	NSC	NSC
Volume (m^3^)			NSC
Length (months)			p < 0.01
Events per decade			NSC

The p-values indicate when there are significant differences between groups.

*NSC* non-significant change.

The five GCMs selected in each project (Supplementary Table S2) cover different ranges of climate sensitivity (black borders in Fig. 6a), and on average, show higher rates of change relative to the multi-model mean of 20 GCMs (circles in Fig. 6b,c). The CMIP5 (CMIP6) subset presents less (greater) differences with respect to the mean of all GCMs (Fig. 6b,c). No differences were found between CHEM/NO CHEM models for both generations of CMIP models (not shown). For example, both CMIP5 CHEM (n = 8) and CMIP5 NO CHEM (n = 12) multi-model averages indicate precipitation reductions of 5%.

### Hydrological projections

**Figure 7:**
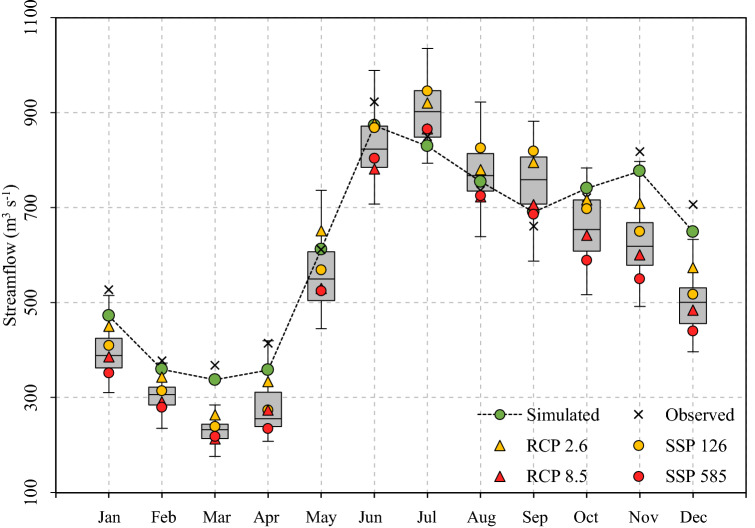
Hydrological projections for the Puelo River basin in the 2040–2070 period. The symbols indicate the multi-model averages under different CMIP5 (RCP 2.6 and 8.5) and CMIP6 (SSP 126 and 585) scenarios (RCP 4.5 and SSP245 not shown for visualization purposes). The green circles (crosses) indicate the simulated (observed) mean streamflows in the reference period (2000–2019).

The different combinations of CMIP projects (2), climate models (5), scenarios (3) and univariate statistical downscaling methods (3) generated 90 possible scenarios for the next five decades (Fig. 2b). The projected average monthly streamflows for the period 2040–2070 indicate that the summer season would be the most affected (− 19% ± 6%), followed by autumn with average reductions of 18% ± 9% (Fig. 7). Consistent with the winter projections in Fig. 6c, mostly neutral trends are expected for winter (Fig. 7). These results are scenario-dependent and present significant differences between CMIP projects for the summer and autumn seasons (Table 3). For example, the CMIP6 and CMIP5 models project decreases of − 22% ± 5% and − 15% ± 6% for the summer season, respectively. In contrast, the three methods of statistical downscaling converge on similar results and did not present significant differences between them (at the 95% confidence level).

**Figure 8:**
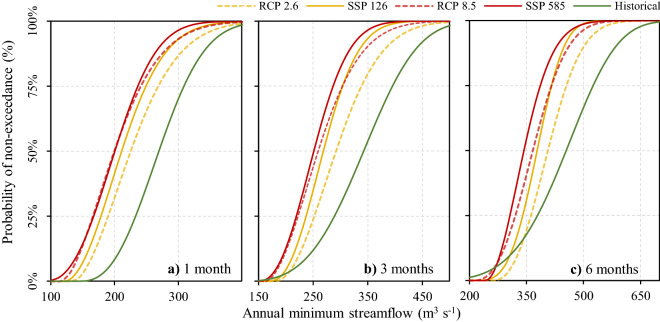
Probability of non-exceedance for minimum annual streamflows of the Puelo River considering time windows of one (**a**), 3 (**b**) and 6 months (**c**). The continuous and discontinuous lines indicate the different scenarios for the CMIP6 and CMIP5 projects, respectively (RCP 4.5 and SSP245 not shown for visualization purposes). The project period is 2020–2070, while the baseline is 1989–2019. The missing data in the historical period were filled with the data modeled by WEAP (Fig. 5).

Independent of the time window analyzed (one to six months), almost all scenarios project increases in the frequency of annual minimum streamflows (Fig. 8). For example, the possibility of experiencing an average streamflow of 250 m^3^s^−1^ for three consecutive months increases from 12 to 35% according to the SSP 126 scenario. The same threshold can reach up to 50% according to the SSP 585 scenario. Only exceptionally low streamflows for six consecutive months (such as the year 2016) did not exhibit a clear increase (Fig. 8c). In these cases, the SSP126 scenario indicated a decrease in dry periods for streamflows less than 300 m^3^s^−1^, while for scenario SSP 585 this threshold was 270 m^3^s^−1^. Overall, the results presented in Fig. 8 show a lower (higher) spread between scenarios for shorter (longer) time windows. For example, the historical annual minimum streamflow associated with a 25% probability of non-exceedance could vary between 56% (RCP 2.6) and 74% (SSP 585) for a time window of one month. The same conditions for six months indicate that the variation could be between 35% (RCP 2.6) and 72% (SSP 585).

**Figure 9:**
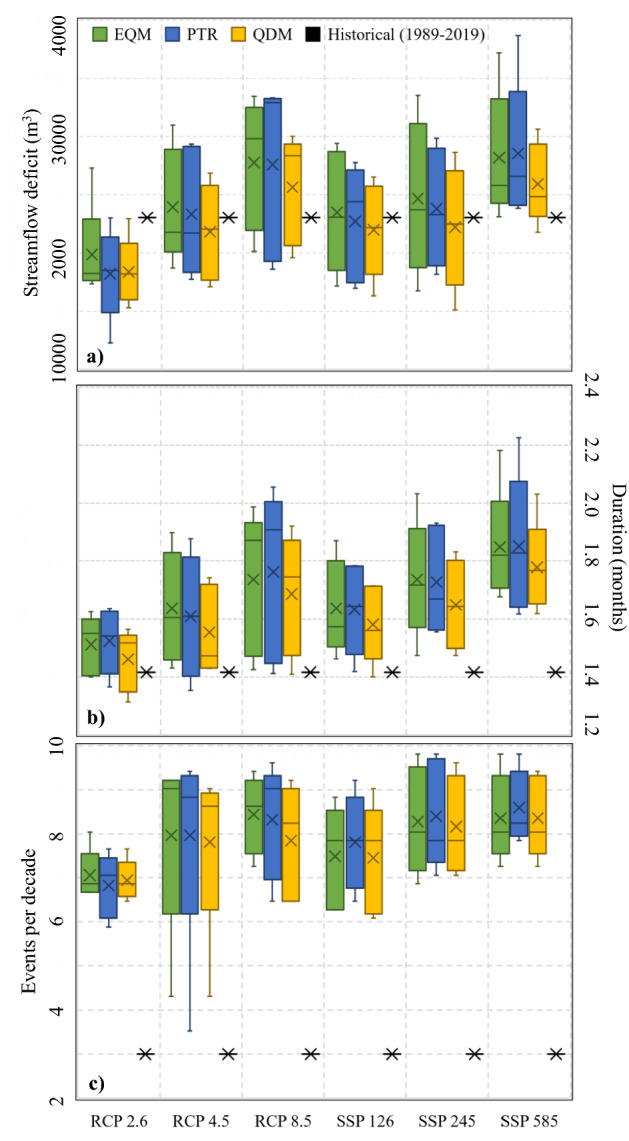
Deficit (**a**), duration (**b**) and frequency (**c**) of events less than 250 m^3^s^−1^ in the period 2020–2070. The results are grouped according to scenarios and downscaling methods. *EQM* empirical quantile mapping. *PTR* power transformation of precipitation. *QDM* quantile delta mapping.

Finally, the frequency, duration and hydrological deficit of severe droughts characterized by Puelo River streamflows below 250 m^3^s^−1^ was analyzed with the objective of anticipating possible hazards that could negatively impact the oceanographic characteristics of the coastal system (Fig. 9). Future scenarios of moderate emissions (SSP 126, 245 and their equivalents), on average, would maintain or slightly decrease the hydrological deficit per event (Fig. 9a). In contrast, the more extreme scenarios (RCP 8.5 and SSP 585) would increase the deficit per event by 20%. The duration of each event would increase slightly in almost all scenarios and methods (1.6 to 1.8 months), except in the RCP 2.6 scenario, where it would remain in the historical range (1.4 months) (Fig. 9b). The number of events per decade is the attribute that would have the greatest changes with respect to the baseline (3 events per decade). In fact, future scenarios project that the number of events could double or triple depending on the greenhouse gas emission scenario (Fig. 9c). The aggregated results by CMIP project showed projections consistent with Figs. 7 and 8. However, only the duration showed a statistically significant difference between CMIP projects (p < 0.01; Table 3). The three downscaling methodologies converged to similar results, with no significant differences between them. However, EQM and PTR tend to amplify the anthropogenic signal slightly, and therefore present greater deficits and durations (Fig. 9).

## Discussion

In recent years there have been increasing reports of climate trends outside the range of natural variability in the Southern Hemisphere^54,55^. The southern Andes have proved to be no exception, since its normally cold and hyper-humid climate showed a clear trend towards warmer and drier conditions since the mid-twentieth century (Fig. 3). Precipitation showed a decreasing pattern mainly during the autumn season, with mean trends of − 8 ± 8% per decade (Fig. 3b). The other seasons converged to values of − 4% per decade, but with variations in their statistical significance. For example, 40% of the stations reported significant decreases during winter (p < 0.05). These values were slightly different from those reported by Boisier et al.^3^, who reported a decrease of 9% per decade for the summer in Chile (1960–2016; 39–48°S). Temperature increased during the whole year, but mainly in summer, when all stations recorded significant trends (0.3 ± 0.1 °C per decade; Fig. 3c). In contrast to the western sector which registered trends of 0.2 °C per decade during the summer, the eastern sector, downwind of the Andean crest, showed trends of 0.4 °C per decade. The heterogeneous trends were consistent with a significant browning of the sub-Antarctic forests in the same area^56^, and with reduced snow cover in the eastern portion (2002–2019; Fig. 4). The decrease in snow cover intensified towards the south, with the basins of the Yelcho, Palena and Aysén Rivers (46°) being the most affected^57^ (Fig. 4b). As a result of the previous trends, streamflow records also showed decreasing trends in the summer and autumn months^28^ (Fig. 3a). Although the maximum trends were concentrated in autumn (− 6 ± 3%), 60% of the fluviometric stations showed statistically significant trends during summer (p < 0.05; Fig. 3a). These results are consistent with those reported in other latitudes of the Andes^2,58,59^, where earlier melting of the snowpack and increased evapotranspiration promote more intense and extensive dry conditions in the summer. Several components of the natural flow regime of the Puelo River also presented significant trends in magnitude, timing and rate of change (p < 0.05; Fig. 3e–g).

Regardless of the model, scenario and CMIP project, the raw climate projections of precipitation and temperature for the next five decades suggest a continuation of warming and drying that have affected the southern Andes in recent decades^1^ (Supplementary Fig. S4 and Fig. 6). Seasonal temperature trends were consistent with instrument records, projecting larger increases during the summer (Figs. 3 and 7). GCMs projected significant declines in summer and autumn precipitation, while the instrumental records only showed a clear signal in autumn (Figs. 3 and 7). However, the decrease in precipitation projected by the models during summer is congruent with the positive SAM trend, which has been attributed primarily to stratospheric ozone depletion^3,60^. Considering the recovery of stratospheric ozone as a result of the Montreal Protocol, it is still uncertain what its impact might be and when it might be reflected in the southern Andes^61^. Although the spatial patterns were similar in both CMIP projects, the projected trends over the period 2040–2070 were significantly higher in the CMIP6 models during the summer and autumn (Fig. 6; Table 3). The causes of higher climate sensitivity in CMIP6 models have been attributed to stronger positive low cloud feedback^24^. In contrast, no significant differences were found between CHEM and NOCHEM models, which is consistent to what was previously reported in the central Andes (37°S)^62^.

The WEAP hydrologic model was employed to simulate hydrological processes in the Puelo River basin. In the sub-basin located near the mouth of the Puelo River (PD in Supplementary Fig. S1), the model reached a KGE of 0.83 and 0.74 for the calibration and validation stages, respectively. Similarly, the model adequately simulated the magnitude and seasonality of SCE and evapotranspiration (Supplementary Fig. S3). Overall, the results were similar to the performance previously reported by Aguayo et al.^26^ and DGA^53^. However, the results are not exempt from different sources of uncertainty. Future studies in the Southern Andes should focus on improving the parameterization of water bodies (e.g. streamflow routing) and infiltration processes (e.g. saturated hydrologic conductivity). Improving the monitoring network would allow the implementation of physically based hydrological models that could validate energy balances that determine the snow melt regime, reducing the biases associated with SCE (Supplementary Fig. S3). Despite current observational limitations (e.g. only 20 years to represent non-stationary conditions), the WEAP model was able to adequately simulate minimum annual streamflows (R^2^ = 0.7) and the frequency, deficit and duration of severe droughts (Fig. 5). Nevertheless, the model failed to capture adequately streamflows with a probability of exceedance of less than 20% (Fig. 5d), where there is a constant dry bias. Beck et al.^63^ estimated that regions characterized by marked altitudinal gradients, low station density and significant solid precipitation present precipitation correction factors greater than 1.5. These factors may be even higher for the southern Andes (> 2.0). Although the integration of instrumental stations with satellite precipitation products (CHIRPSv2; Table 1) managed to reduce the bias, it is essential to increase monitoring in the Southern Andes. This would allow optimal evaluation of regional climate models, dynamic downscaling methodologies, and consistency with linear orographic parameterizations.

The 90 scenarios generated from different combinations of CMIP projects (n = 2), models (n = 5), scenarios (n = 3) and univariate statistical downscaling methods (n = 3) were consistent in predicting significant changes at different time scales. The projected average monthly streamflows for the period 2040–2070 indicate that the summer (− 19% ± 6%) and autumn periods would be the most affected (− 18% ± 9%; Fig. 7). Despite the methodological differences with Aguayo et al.^26^ (time period, downscaling method, conceptual hydrological model, among others), the results of both studies were consistent in the magnitude and seasonality of the projected changes, with slight differences in autumn (− 12%) and winter (− 9%). The differences between both studies for winter could be attributed to the different modules of snow melting (e.g. the WEAP model considers two threshold temperatures for the degree-day method). In relation to the rest of the basin modeled by the National Water Balance^53^ (36–46°S), the Puelo river basin presented more favorable annual hydrological projections (ΔQ < 10%).

The analysis of frequency showed that almost all scenarios and time windows project increases in the frequency of annual minimum streamflows (Fig. 8). Only exceptionally low streamflows for six consecutive months (such as the year 2016) did not exhibit a clear increase (Fig. 8c). In this case, the SSP585 scenario indicated a decrease in dry periods for streamflows less than 270 m^3^s^−1^. These projections are consistent with the increased frequency of severe droughts (Q < 250 m^3^s^−1^; 95% probability of exceedance) that could negatively impact the physical–chemical characteristics of coastal systems (Fig. 9a). These events would be characterized by slight variations in the hydrological deficit and duration of each event, depending on the scenario, CMIP project and downscaling method (Fig. 9b,c). For example, according to the SSP585 scenario, the deficit would increase from 2300 m^3^ to 2750 ± 490 m^3^. The same scenario projects that the duration would change from 1.4 to 1.8 ± 0.2 months. These results are consistent with those reported by Cook et al.^1^, who determined that the southern Andes could be one of the regions where the greatest increases in severe droughts would occur under the new CMIP6 models.

Past evaluations have shown that the hydrological differences between the CMIP3 and CMIP5 models depend on the region, scenario, and hydrological regime, among other factors^58,64,65^. For example, unlike Demaria et al.^58^, who found no significant hydrological and climatic variations between CMIP3 and CMIP5 models in the central Andes (Table 2), in the present study the disaggregation of GCMs by CMIP project indicates significantly different results in summer and autumn seasons (Figs. 6–9; Table 3). It is recommended that past/future climate impact assessments update/adapt the new simulations and scenarios, as more CMIP6 models become available. In this study, all GCMs are assigned equal weights to simulate the hydrological impacts of climate change, assuming that GCMs are independent of each other, which may not be true due to the common modules and parameterizations among some GCMs^66^. Thus, the equal weighting may not be optimal for multi ensembles and should be addressed by future studies.

The three downscaling methodologies converged to similar results, with no significant differences between them. However, EQM and PTR tended to amplify the anthropogenic signal slightly, and therefore presented greater deficits and durations (Fig. 9). These differences could be explained by the fact that EQM assumes that the function of error correction values found in a calibration period can be applied to any time period of interest. This stationarity assumption is responsible for altering the raw model projections^67^. In contrast, QDM multiplies observed values by the ratio of the modeled values in the same quantiles, hence it is not constrained by the stationarity assumption^47^. It is important to mention that Aguayo et al.^26^ had previously used the delta-change method, finding that the probability of occurrence of extreme events (e.g. year 2016) could be doubled in the near future (2030–2060). These results differ from those reported in this study, where the probability of such extreme events (Q < 300 m^3^s^−1^ during six consecutive months) does not increase. Considering these antecedents, the underlying assumption in the delta-change method (maintenance of natural variability) should be studied carefully. In this study, the variability of the climatic anomalies, reflected in the standard deviation of the raw values, remained constant (Supplementary Fig. S5), suggesting that future droughts could be caused by an overlap of projected average conditions.

The climate and hydrological projections reported in this study are a grim prospect for the coastal system of the southern Andes and for the various productive activities that take place there. The coastal sites of the southern Andes host much of the national salmon farming and mytilid culture. According to FAO Global Fishery and Aquaculture Production Statistics, Chile currently leads the industry, being the second exporter of salmon and trout and the first exporter of mussels. Soto et al.^22^ found a high dependence of coastal salmon farming on freshwater input; the increased vulnerability of this activity is strongly associated with hydro-climatic projections. According to these authors, coastal salmon farming that takes place in systems influenced by high freshwater input contributes around 32% of the total production of Chilean salmon farming. Similarly, mytilid culture has also shown to be highly sensitive to extreme hydro-climatic events. According to the experience of local producers and the results of the Monitoring and Surveillance Program of the Fisheries Development Institute (IFOP) on the availability of larval mitylids in the internal sea of Chiloé, coastal sites with high freshwater input and presence of natural banks ensure excellent levels of capture and a high probability that the seeds are mussels. In this sense, the increase in severe droughts that could exceed critical thresholds linked to anomalous oceanographic events is of great concern^19,21,31^ (Fig. 9).

Finally, in light of the reported results and possible environmental, social and economic consequences, the transferability of water to lower latitudes in the Andes should carefully evaluate the hydro-climatic synergies projected for the coming decades in an area where freshwater inputs play an important environmental role^68^. In addition, the results of the study suggest the need to increase the density and quality of meteorological stations in the high areas of the southern Andes. It is also necessary to consider possible synergies with other anthropogenic threats such as land cover change and the increase of forest fires. These interactions could significantly impact the quality and hydrological regime of the basins, so future conservation plans based on scientific evidence could include measures to mitigate the environmental consequences of a warmer and drier climate in the southern Andes.

## Supplementary Information

Supplementary Information.

## Data Availability

The CMIP5 and CMIP6 multi-model databases are available thanks to the World Climate Research Project (WCRP; https://esgf-node.llnl.gov/search/esgf-llnl). The observed data of precipitation, temperature and streamflow were obtained from the General Directorate of Water (DGA; https://dga.mop.gob.cl) and the Meteorological Service (DMC; http://www.meteochile.cl) in Chile, and from the Undersecretary of Water Resources (SRHA; https://snih.hidricosargentina.gob.ar) and the Meteorological Information Center (SMN; https://www.smn.gob.ar) in Argentina. The bias corrected datasets analyzed during the current study are available from the corresponding author on reasonable request.
