# Supplementary material for: Hydrological droughts in the southern Andes (40–45°S) from an ensemble experiment using CMIP5 and CMIP6 models
Source: Sci Rep. 2021 Mar 9;11:5530. doi: 10.1038/s41598-021-84807-4 (PMC7943561; doi:10.1038/s41598-021-84807-4)
Supplement: Supplementary file 1 — Supplementary Information. [file 41598_2021_84807_MOESM1_ESM.docx]

Supplementary Material for: Scientific Report

Hydrological droughts in the southern Andes (40-45ºS) from an ensemble experiment using CMIP5 and CMIP6 models

Rodrigo Aguayo^1^, Jorge León-Muñoz^2,3,*^, René Garreaud^4,5^, Aldo Montecinos^6,7^

^1^ Centro EULA, Facultad de Ciencias Ambientales, Universidad de Concepción, Concepción, Chile.

^2^ Departamento de Química Ambiental, Facultad de Ciencias, Universidad Católica de la Santísima Concepción, Concepción, Chile.

^3^ Centro Interdisciplinario para la Investigación Acuícola (INCAR), Concepción, Chile

^4^ Departamento de Geofísica, Facultad de Ciencias Físicas y Matemáticas, Universidad de Chile, Santiago, Chile

^5^ Centro de Ciencia del Clima y la Resiliencia (CR2), Santiago, Chile

^6^ Departamento de Geofísica, Facultad de Ciencias Físicas y Matemáticas, Universidad de Concepción, Concepción, Chile.

^7^ Centro de Recursos Hídricos para la Agricultura y Minería (CRHIAM), Concepción, Chile.

Corresponding author: Jorge León-Muñoz, jleon@ucsc.cl, Tel. +56 41 2345102.


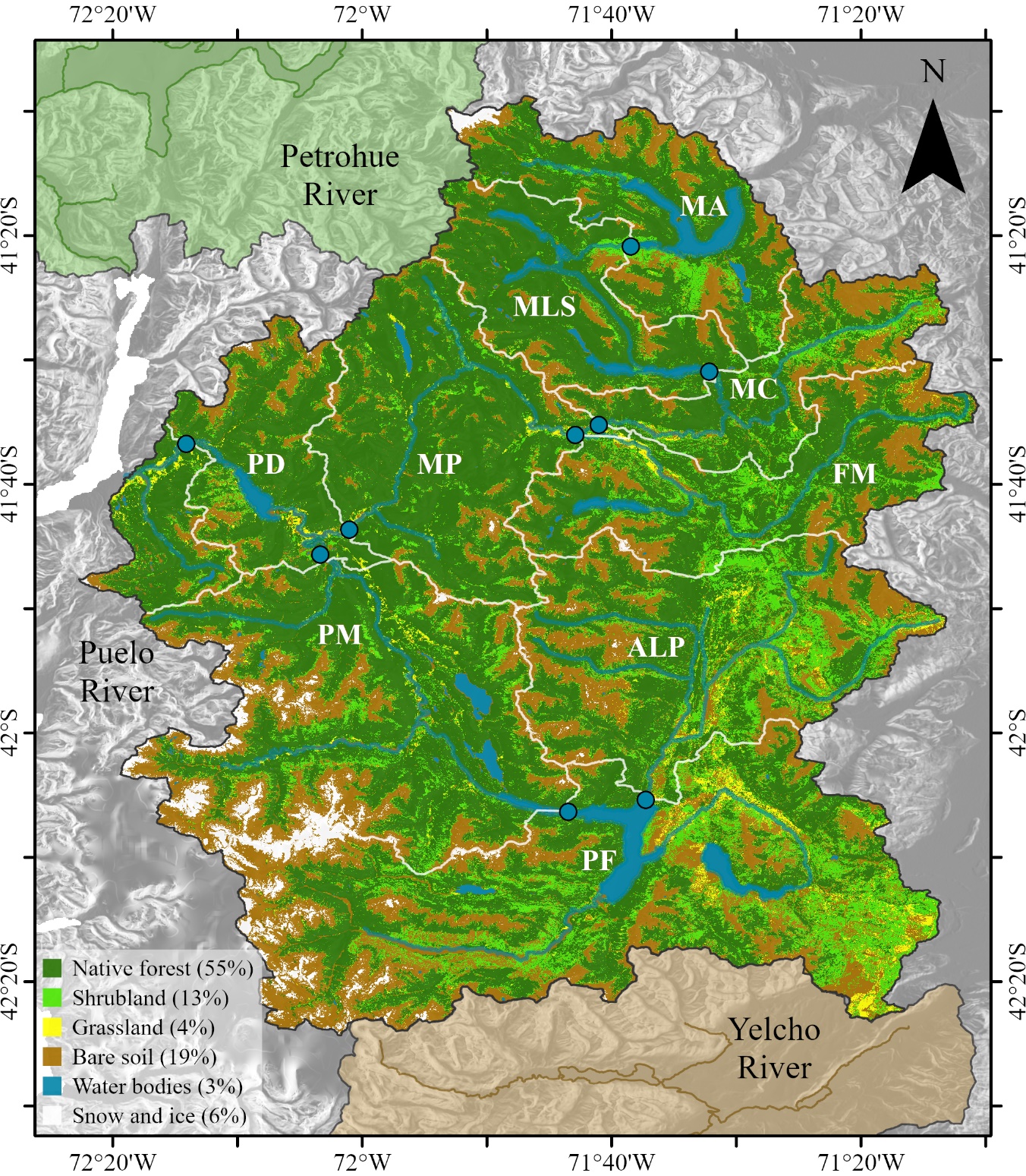


# Figure S1: Land cover of the Puelo River basin in the year 2001 (overall accuracy of 85.3%). The legend indicates the percentages of each land cover class. The blue circles indicate the fluviometric stations used in hydrological modeling. The acronyms show the name of each sub-basin generated by the stations. The figure was generated using ArcGIS Pro 2.7.0 (https://www.esri.com/en-us/arcgis/products/arcgis-pro/).


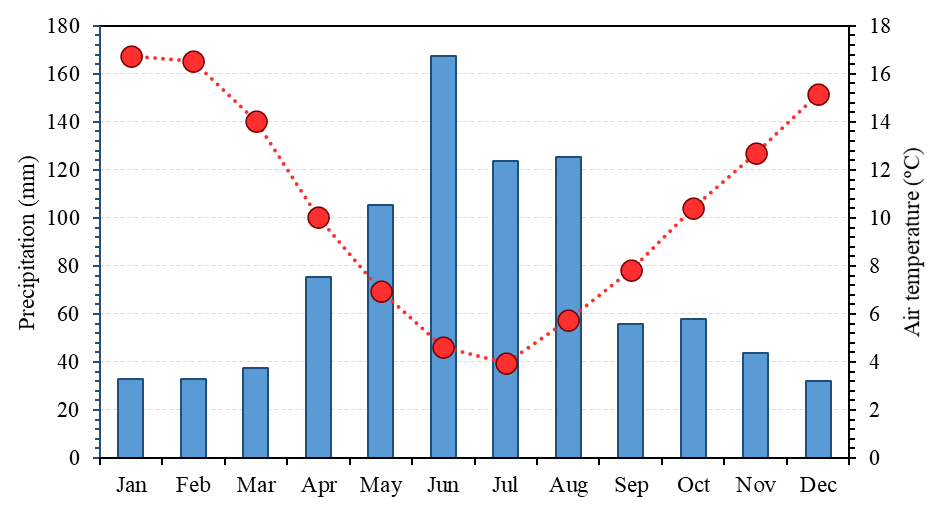


MAM

25%

DJF

11%

JJA

47%

SON

18%

# Figure S2: Climograph of the station "El Bolson" in the period 1982-2018. The location of the station is shown in Fig. 1.


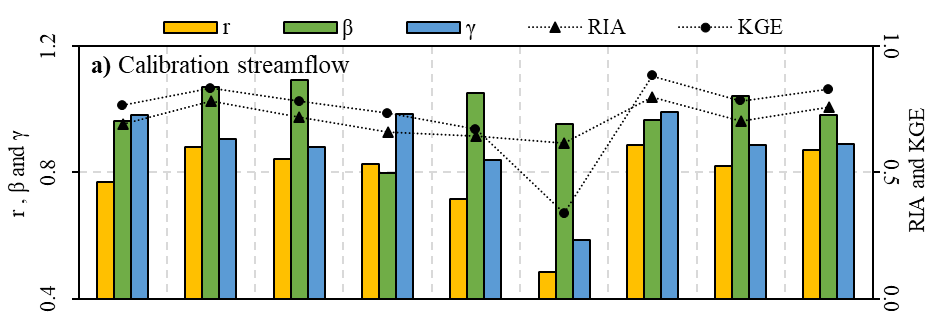

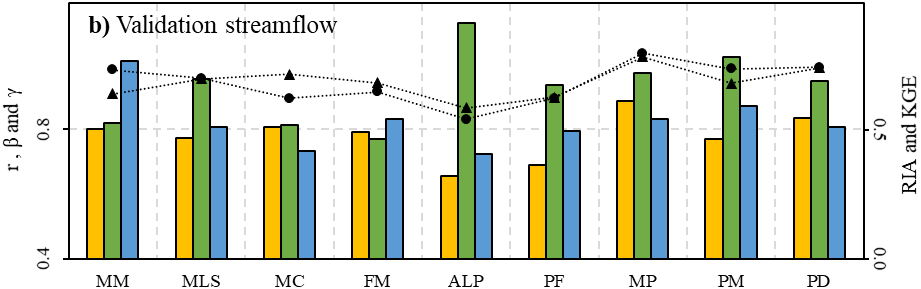

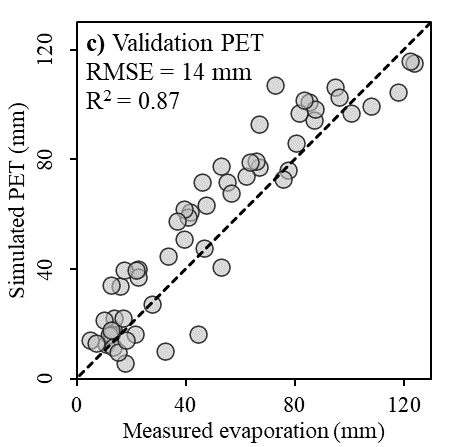


1:1


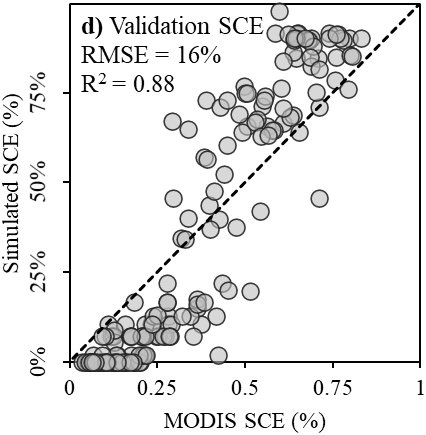


1:1

# Figure S3: Performance of the WEAP hydrological model during the calibration (a) and validation (b) stages for the nine sub-basins of the Puelo River basin. Performance indices are the Modified Kling–Gupta efficiency (KGE) and the Refined Index of Agreement (KGE). The KGE index is based on the Pearson correlation (r), bias (β; mean ratio) and the variability (γ; coefficient of variation ratio). Validation of Potential Evapotranspiration (PET; c) and Snow Cover extent (SCE; d). PET and SCE were validated with an evaporimeter (Fig. 1; 2002-2012) and MODIS satellite data (2002-2019), respectively.


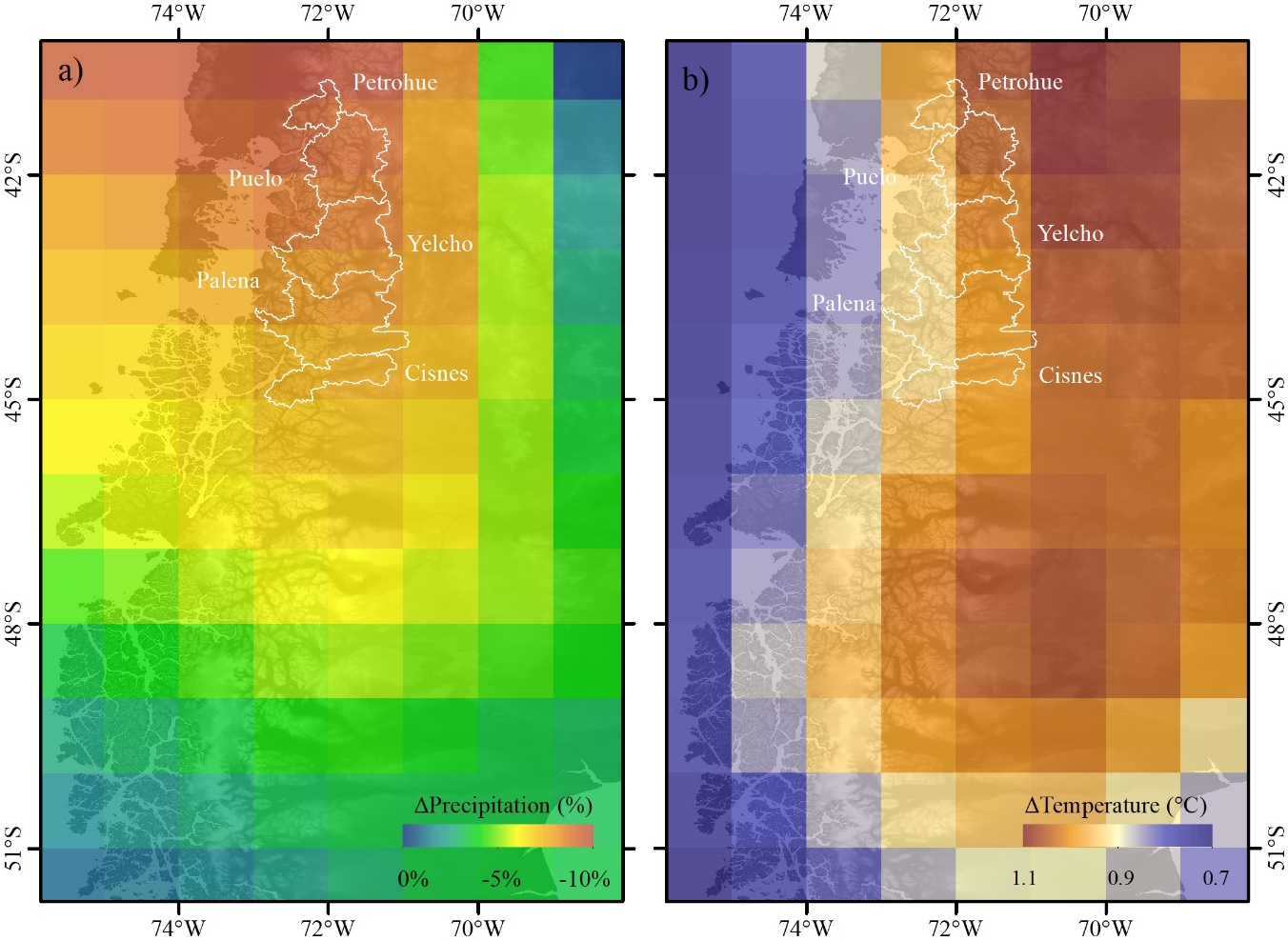


# Figure S4: Multi-model mean of precipitation (a) and temperature (b) from the CMIP6 project for the period 2040-2070 considering 2001-2018 as reference. The multi-model mean considers the three scenarios of the 20 GCMs (SSP 126, 245 and 585). The figure was generated using ArcGIS Pro 2.7.0 (https://www.esri.com/en-us/arcgis/products/arcgis-pro/).


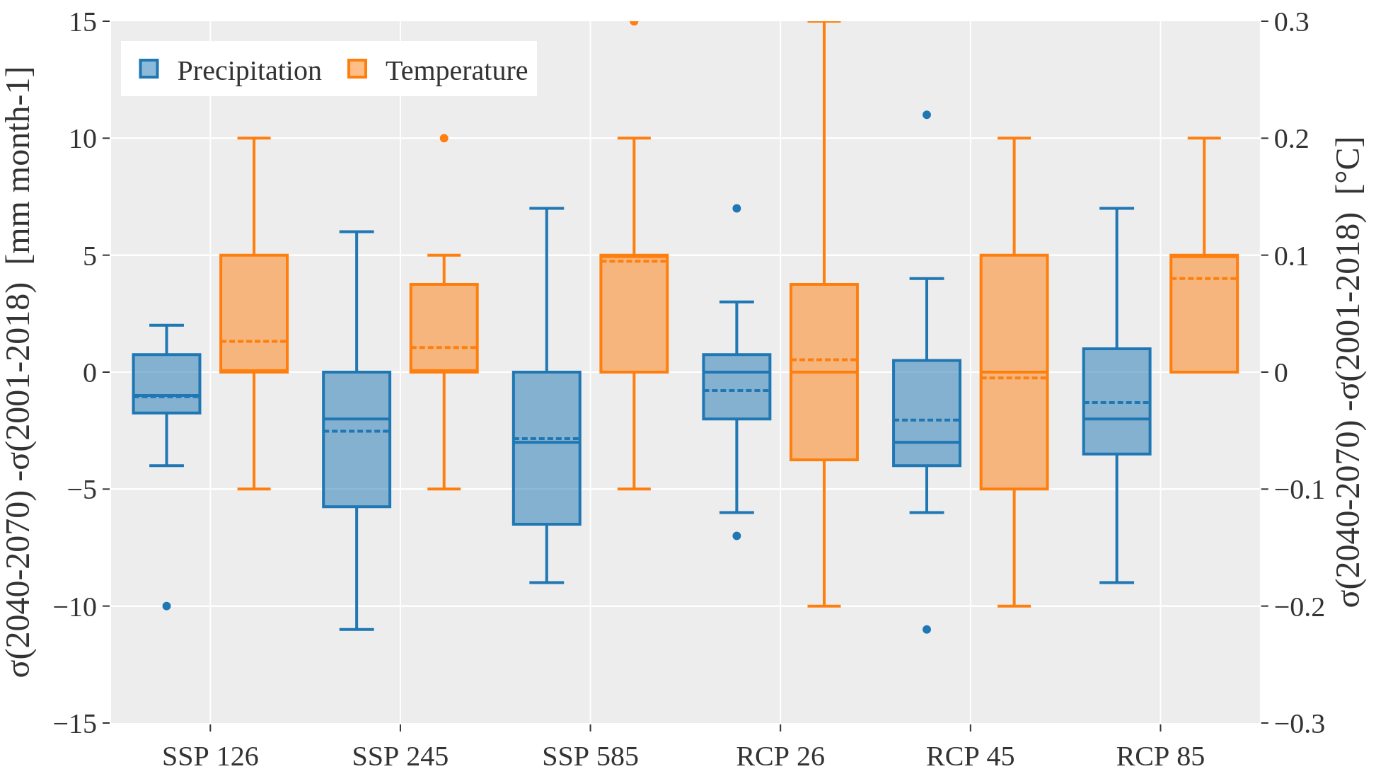


# Figure S5: Comparison of the variability of precipitation and temperature anomalies for the base (2001-2018) and projected period (2040-2070). Anomalies extracted from the annual cycle of each period. Raw data from 20 CMIP5 GCMs and 20 CMIP6 GCMs.

# Text S1: WEAP calibration process

The calibration of the WEAP hydrological model is an iterative process. The **first step** adjusts the parameters that vary among sub-basins and land cover level (Table S1), using the period 2000-2010 as reference (2009-2014 for ALP, PF and FM sub-basins). The parameters that vary depending on the land cover, that is, one parameter per land cover for the entire basin, are obtained according to different methodologies, which are detailed below:

- Runoff Resistance Factor (RRF, Table S1): RRF is calculated for each land cover from the Leaf Area Index (LAI), since there is a proportionality relationship for the generation of surface runoff^1^. The LAI is obtained from the product MOD15A2H for the same date of the land cover classification, in order to maintain temporal consistency. Finally, the RRF parameters are standardized under an additional parameter, which is then part of the calibration process (Table S1).
- Crop Coefficient (K_c_, Table S1): Kc is obtained for each land cover according to the methodology proposed by Hunink et al.^2^, and widely used in other hydrological models^3^. The equation to calculate the crop coefficient from NDVI is as follows:

$$\mathrm{kc}_{t} = \mathrm{kc}_{\min}+ \left( \mathrm{kc}_{\max} +\mathrm{kc}_{\min} \right) \cdot\frac{\mathrm{NDVI}_{t} - \mathrm{NDVI}_{\min}}{\mathrm{NDVI}_{\max} - \mathrm{NDVI}_{\min}}$$

where kc_t_ is the crop coefficient for a given moment in time, NDVI_t_ is the NDVI observed, NDVI_min_ is the NDVI value of bare soil (-0.1), NDVI_max_ is the maximum NDVI value in the area (1.0). The coefficients kc_min_ and kc_max_ are respectively the minimum and maximum values for the crop coefficient in the area (kc_min_= 0.4 and kc_max_= 1.2). The Kc obtained from this methodology for each land cover are the following: native forest (1.1), shrubland (0.96), grassland (0.91), bare soils (0.65), snow and ice (0.39). For water bodies we assume potential evapotranspiration (e.g. kc = 1).

- Albedo Lower Bound (A_1_, Table S1): Minimum albedo values are obtained from Hartmann^4^, assuming typical values for each land cover. WEAP calculates the albedo at each time step assuming a linear function between snow accumulation and lower and upper albedo limits^1^.

The parameters that are constant in the basin, such as the upper albedo bound and the freezing and melting temperatures (Table S1), are obtained directly from the PEST (Independent Parameter Estimation and Uncertainty Analysis) calibration process, considering the nine sub-basins as an objective function.

In the **second step**, the parameters specific to each sub-basin (Table S1) are calibrated from upstream to downstream of the basin, considering only the associated fluviometric station as an objective function. It should be noted that the Cloud Fraction parameter is calculated directly from the MODIS MOD06_L2 product for each month and sub-basin.

Once an iteration has been completed, the performance indices are compared with those obtained in the previous iteration to analyze the significance of the changes made. The calibration of the hydrological model is completed when there are no significant changes between iterations. Then the model is validated for subsequent years (2011-2019; 2015-2019 for the PFA, FP and FM sub-basins).

# **Table S1:** Parameters of the hydrological model WEAP

| **Parameter** | **Module** | **Variation** | **Range / unit** | **Calibration** |
| --- | --- | --- | --- | --- |
| Runoff resistance factor | Runoff  and PET | Land cover | 0 - 10 | MOD15A2H  Semi-automatic |
| Crop coefficient |  |  | 1. - 1.0 | Hunink et al. (2017) |
| Albedo lower bound | Snow |  | 0.0 – 0.7 | Hartmann (2015) |
| Albedo upper bound |  | Basin | 0.7 - 1.0 | Automatic |
| Melting point |  |  | 0°C - 10°C | Automatic |
| Freezing point |  |  | -20°C - 0°C | Automatic |
| Capacity | Root  zone | Sub-basin | > 0 mm | Automatic |
| Conductivity |  |  | > 0 mm month^-1^ | Automatic |
| Preferred flow direction |  |  | 0.0 - 1.0 | Automatic |
| Capacity | Deep  zone |  | > 0 mm | Automatic |
| Conductivity |  |  | > 0 mm month^-1^ | Automatic |
| Cloud fraction | PET and snow |  | 0 - 100% | MODIS MOD06_L2 |

# **Table S2:** General Circulation Models (GCMs) of the CMIP5 and CMIP6 projects. The CHEM models have interactive ozone through a coupled chemical climate model. In contrast, the NOCHEM CMIP5 (CMIP6) models have one prescribed time-varying ozone path for all (each) scenarios. This information was obtained from Eyring et al.^5^ and Keeble et al.^6^. Asterisks indicate the models selected for statistical downscaling. The CESM2 model prescribes ozone values from the CESM2-WACCM model. Lon-Lat columns are the spatial resolution of each model output (Longitude and Latitude).

| **CMIP5 models** | | | | **CMIP6 models** | | | |
| --- | --- | --- | --- | --- | --- | --- | --- |
| **Model** | **Lon - Lat** | | **Ozone** | **Model** | **Lon - Lat** | | **Ozone** |
| BCC-CSM1.1-m | 1.1 | 1.1 | NOCHEM | ACCESS-CM2 | 1.9 | 1.3 | NOCHEM |
| CanESM2 | 2.8 | 2.8 | NOCHEM | ACCESS-ESM1-5 | 1.9 | 1.3 | NOCHEM |
| CCSM4 | 1.3 | 0.9 | CHEM | BCC-CSM2-MR | 1.1 | 1.1 | NOCHEM |
| CESM1-CAM5 | 1.3 | 0.9 | CHEM | CAMS-CSM1-0 | 1.1 | 1.1 | NOCHEM |
| CESM1-WACCM | 2.5 | 1.9 | CHEM | CanESM5* | 2.8 | 2.8 | NOCHEM |
| CNRM-CM5 | 1.4 | 1.4 | CHEM | CESM2-WACCM* | 1.3 | 0.9 | CHEM |
| CSIRO-Mk3.6.0 | 1.9 | 1.9 | NOCHEM | CESM2* | 1.3 | 0.9 | NOCHEM |
| EC-EARTH* | 1.1 | 1.1 | NOCHEM | FGOALS-f3-L | 1.3 | 1.0 | NOCHEM |
| GFDL-CM3 | 2.5 | 2.0 | CHEM | FIO-ESM-2-0 | 1.3 | 0.9 | NOCHEM |
| GISS-E2-H | 2.5 | 2.0 | NOCHEM | GFDL-ESM4 | 1.3 | 1.0 | CHEM |
| GISS-E2-R | 2.5 | 2.0 | NOCHEM | INM-CM4-8 | 2.0 | 1.5 | NOCHEM |
| HadGEM2-AO* | 1.9 | 1.3 | NOCHEM | INM-CM5-0 | 2.0 | 1.5 | NOCHEM |
| HadGEM2-ES | 1.9 | 1.3 | NOCHEM | IPSL-CM6A-LR* | 2.5 | 1.3 | NOCHEM |
| IPSL-CM5A-MR* | 2.5 | 1.3 | CHEM | KACE-1-0-G | 1.9 | 1.3 | NOCHEM |
| MIROC5 | 1.4 | 1.4 | NOCHEM | MIROC6* | 1.4 | 1.4 | NOCHEM |
| MPI-ESM-LR* | 1.9 | 1.9 | NOCHEM | MPI-ESM1-2-LR | 1.9 | 1.9 | NOCHEM |
| MPI-ESM-MR* | 1.9 | 1.9 | NOCHEM | MRI-ESM2-0 | 1.1 | 1.1 | CHEM |
| MRI-CGCM3 | 1.1 | 1.1 | NOCHEM | NESM3 | 1.9 | 1.9 | NOCHEM |
| NorESM1-M | 2.5 | 1.9 | CHEM | NorESM2-LM | 2.5 | 1.9 | CHEM |
| NorESM1-ME | 2.5 | 1.9 | CHEM | NorESM2-MM | 1.3 | 0.9 | CHEM |

# References

1. Yates, D., Sieber, J., Purkey, D. & Huber-Lee, A. WEAP21 - A demand-, priority-, and preference-driven water planning model. Part 1: Model characteristics. *Water Int.* **30**, 487–500 (2005).

2. Hunink, J. E. *et al.* Hydrological modelling using satellite-based crop coefficients: A comparison of methods at the basin scale. *Remote Sens.* **9**, (2017).

3. Terink, W., Lutz, A. F., Simons, G. W. H., Immerzeel, W. W. & Droogers, P. SPHY v2.0: Spatial Processes in HYdrology. *Geosci. Model Dev.* **8**, 2009–2034 (2015).

4. Hartmann, D. L. *Global physical climatology: Second Edition*. *Global Physical Climatology: Second Edition* (2015). doi:10.1016/C2009-0-00030-0.

5. Eyring, V. *et al.* Long-term ozone changes and associated climate impacts in CMIP5 simulations. *J. Geophys. Res. Atmos.* **118**, 5029–5060 (2013).

6. Keeble, J. *et al.* Evaluating stratospheric ozone and water vapor changes in CMIP6 models from 1850-2100. *Atmos. Chem. Phys.* 1–68 (2020) doi:10.5194/acp-2019-1202.
